# Pemafibrate therapy increases serum zinc levels in patients with metabolic dysfunction–associated steatohepatitis: a prospective study

**DOI:** 10.1186/s12876-026-04806-5

**Published:** 2026-04-09

**Authors:** Noritaka Wakui, Yu Ogino, Hideki Nagumo, Naho Watanabe, Kunihide Mouri, Naoyuki Yoshimine, Kojiro Kobayashi, Takanori Mukozu, Teppei Matsui, Takahisa Matsuda

**Affiliations:** Division of Gastroenterology and Hepatology, Department of Internal Medicine (Omori), School of Medicine, Faculty of Medicine, Toho University, 6-11-1 Omori-nishi, Ota-ku, Tokyo, 143-8541 Japan

**Keywords:** Metabolic dysfunction-associated steatohepatitis, Metabolic dysfunction-associated steatotic liver disease, Pemafibrate, Hypertriglyceridemia, Zinc, PPARα modulator

## Abstract

**Background:**

Zinc deficiency is common in chronic liver disease and is closely associated with the progression of hepatic fibrosis and metabolic dysfunction. Although pemafibrate, a selective peroxisome proliferator-activated receptor alpha modulator, has had beneficial effects on lipid metabolism, inflammation, and oxidative stress, its impact on zinc metabolism in patients with metabolic dysfunction-associated steatohepatitis (MASH) remains unclear.

**Methods:**

This prospective study involved patients with biopsy-confirmed MASH complicated by hypertriglyceridemia (fasting triglycerides ≥ 150 mg/dL) who were treated with pemafibrate (0.2 mg twice daily). Serum zinc levels were evaluated at baseline and at 6, 12, 24, and 36 months after initiation of treatment. Subgroup analyses were performed according to cirrhosis status and baseline serum zinc level (< 80 µg/dL vs. ≥80 µg/dL). Changes in liver-related biochemical parameters and albumin levels were also assessed.

**Results:**

Twenty-six patients (mean age 56.3 ± 12.6 years; 17 men) were followed for 36 months. Serum zinc levels had increased significantly at 6 months after initiation of pemafibrate and remained significantly elevated through to 36 months. Patients without cirrhosis showed a sustained increase in serum zinc levels throughout the observation period, whereas those with cirrhosis had a delayed and transient response. The serum zinc level increased earlier in patients with zinc deficiency at baseline than in those with a preserved level at baseline. Serum albumin levels increased significantly up to 24 months but not up to 36 months, whereas serum zinc levels remained elevated, suggesting the involvement of albumin-independent mechanisms. Pemafibrate therapy was also associated with significant improvements in triglycerides, liver enzymes, and fibrosis-related markers.

**Conclusions:**

Pemafibrate therapy was associated with a significant and sustained increase in the serum zinc level in patients with hypertriglyceridemia-related MASH, even in the absence of zinc supplementation. However, given the small sample size, the absence of a control group, and the single-arm study design, future large-scale multicenter studies that include a greater number of patients are warranted.

## Background

Zinc is an essential trace element that is required for the activity of numerous enzymes and the maintenance of cellular structure, playing important roles in human growth, immune function, and antioxidant defense mechanisms [1]. Zinc deficiency has a wide range of clinical manifestations, including growth retardation, fatigue, dermatological disorders, neurological impairment, and impaired immune responses [2]. Zinc is primarily absorbed in the duodenum and upper small intestine, with excess zinc being secreted into the gastrointestinal tract via pancreatic fluid and excreted in the feces together with unabsorbed zinc.

Chronic liver disease is frequently associated with zinc deficiency [3]. Hepatic fibrosis worsens as the liver disease progresses and is accompanied by a gradual decline in the serum zinc level [4]. Zinc deficiency reduces the free radical-scavenging activity of circulating metallothionein, leading to increased oxidative stress and the persistence of hepatic inflammation, suppression of apoptosis, an increased risk of hepatocellular carcinoma, and progression of hepatic fibrosis [5, 6]. The decline in systemic zinc levels with progression of chronic liver disease and hepatic fibrosis accelerates fibrogenesis and increases the risk of hepatocellular carcinoma, which creates a vicious cycle. Therefore, zinc deficiency is an important clinical concern in patients with chronic liver disease.

Nonalcoholic fatty liver disease (NAFLD) is the most prevalent form of chronic liver disease worldwide, affecting approximately 25% of the global population [7, 8]. The prevalence of zinc deficiency is higher in patients with NAFLD than in healthy individuals and is even higher in patients with nonalcoholic steatohepatitis (NASH) compared with those with simple steatosis [9, 10]. Moreover, zinc deficiency has been reported to induce mitochondrial oxidative stress in the liver of patients with NASH, thereby promoting iron overload, exacerbating insulin resistance, and accelerating hepatic fibrosis [11]. Accordingly, zinc status is an important clinical consideration in NAFLD and NASH.

In 2023, a new disease nomenclature was proposed whereby NAFLD was renamed metabolic dysfunction-associated steatotic liver disease (MASLD) and NASH was renamed metabolic dysfunction-associated steatohepatitis (MASH) [12]. In line with this updated framework, patients with hepatic steatosis who satisfy at least one metabolic risk factor (abnormally high body mass index or waist circumference, hyperglycemia, elevated blood pressure, hypertriglyceridemia, or dyslipidemia) are classified as having MASLD, while those with steatohepatitis are classified as having MASH. Subsequent studies have demonstrated that approximately 98%–99% of patients previously diagnosed with NAFLD/NASH fulfill the diagnostic criteria for MASLD/MASH and that the clinical characteristics and prognostic outcomes in reclassified patients are similar to those of conventional NAFLD/NASH [13–15]. Accordingly, the terms NAFLD and MASLD, as well as NASH and MASH, are now considered essentially interchangeable in clinical practice.

Importantly, MASLD/MASH is strongly associated with metabolic and dietary risk factors, including obesity, insulin resistance, and excessive intake of saturated fat and fructose-rich sugars. A high-fat diet primarily promotes hepatic steatosis, whereas excessive fructose intake increases de novo lipogenesis, ATP depletion, and uric acid production, leading to mitochondrial oxidative stress and lipid accumulation in the liver. Notably, a high intake of fat combined with a high intake of sugar has been shown to synergistically accelerate the progression from simple steatosis to steatohepatitis, which is characterized by hepatic inflammation, oxidative stress, and fibrogenesis [16, 17]. These pathophysiological processes of insulin resistance, oxidative stress, inflammatory cytokine activation, and fibrogenesis are central to the progression of MASLD to MASH. In this context, zinc deficiency may further exacerbate these mechanisms by impairing antioxidant defense systems and promoting persistent inflammation, thereby contributing to disease progression.

Therefore, assessment of zinc status remains clinically relevant in patients with MASLD/MASH. Hypertriglyceridemia plays a central role in the pathogenesis of the hepatic steatosis observed in most patients with MASLD. One of the therapeutic agents that targets hypertriglyceridemia is pemafibrate, a selective peroxisome proliferator-activated receptor alpha (PPARα) modulator. Pemafibrate is the first selective PPARα modulator developed to selectively activate transcription of PPARα target genes. In a Phase III clinical trial, the triglyceride-lowering efficacy and the hepatic and renal safety profiles of pemafibrate were superior to those of fenofibrate in patients with dyslipidemia [18]. Honda et al. reported that pemafibrate attenuates the pathogenesis of NASH by modulating lipid turnover and energy metabolism in the liver [19]. Furthermore, pemafibrate has been shown to exert anti-inflammatory effects and to reduce oxidative stress [20].

Despite these beneficial effects, the impact of pemafibrate on serum zinc levels remains unclear. Therefore, we conducted this study to investigate the effects of pemafibrate therapy on serum zinc concentrations in patients with MASH complicated by hypertriglyceridemia.

## Materials and methods

### Enrollment of the participants

Patients who underwent liver biopsy at Toho University Omori Medical Center between July 2021 and December 2023 and were diagnosed with NASH were prospectively enrolled in this study if they had a fasting triglyceride level of ≥ 150 mg/dL. The inclusion criteria were age ≥ 20 years and willingness to receive pemafibrate therapy for hypertriglyceridemia. NAFLD was diagnosed in accordance with the most recent guidelines published by the American Association for the Study of Liver Diseases [21] based on the following criteria: hepatic steatosis detected by imaging; absence of significant alcohol consumption (ethanol intake < 210 g/week for men and < 140 g/week for women); exclusion of other causes of hepatic steatosis; and absence of chronic liver disease with a defined etiology, including viral hepatitis (B or C), primary biliary cholangitis, or autoimmune hepatitis. In accordance with the revised disease classification proposed in 2023 whereby NAFLD was redefined as MASLD [12], patients with hepatic steatosis who satisfied at least one of the following metabolic criteria were classified as having MASLD: body mass index ≥ 23 (calculated as kg/m²) or waist circumference > 94 cm in men and > 80 cm in women; fasting plasma glucose ≥ 100 mg/dL, 2-h postprandial glucose ≥ 140 mg/dL, glycated hemoglobin (HbA_1c_) ≥ 5.7%, or ongoing treatment for type 2 diabetes mellitus; blood pressure ≥ 130/85 mmHg or use of antihypertensive medication; a serum triglyceride level ≥ 150 mg/dL or treatment for dyslipidemia; and serum high-density lipoprotein cholesterol ≤ 40 mg/dL in men and ≤ 50 mg/dL in women or treatment for dyslipidemia. Patients who satisfied at least one of these criteria were included in the study.

The exclusion criteria were as follows: (a) previous or ongoing treatment with pemafibrate; (b) severe hepatic impairment or biliary obstruction; (c) renal dysfunction (defined as serum creatinine ≥ 2.5 mg/dL or creatinine clearance < 40 mL/min); (d) presence of gallstones; (e) pregnancy or breastfeeding; (f) history of surgery involving the duodenum or small intestine; and (g) current use of zinc supplementation. Criteria (b) through (e) were applied in accordance with the contraindications for pemafibrate therapy. Patients with a history of surgery involving the duodenum or small intestine were excluded because these regions are the primary sites of zinc absorption.

The study protocol was approved by the ethics committee of Toho University (Approval no. M23242 21002) and conducted in accordance with the Declaration of Helsinki. Written informed consent was obtained before enrolment in the study.

### Administration of pemafibrate and laboratory assessments

All patients received oral pemafibrate 0.2 mg twice daily. Follow-up visits were scheduled every 3 months during the treatment period, and patients were observed for a total of 36 months. Changes in the serum zinc level after initiation of pemafibrate therapy were evaluated in the entire cohort and in subgroups according to cirrhosis status (present/absent) and baseline serum zinc level (< 80 µg/dL or ≥ 80 µg/dL). Cirrhosis was defined by histopathological findings of stage F4 fibrosis on liver biopsy or a Fibrosis-4 index score > 2.67 [22]. Zinc deficiency was defined as a serum zinc concentration of < 80 µg/dL in accordance with the Japanese clinical practice guidelines for zinc deficiency [23]. The following laboratory parameters were also measured: aspartate aminotransferase, alanine aminotransferase, albumin, gamma-glutamyl transferase, total bilirubin, platelet count, high-density lipoprotein cholesterol, low-density lipoprotein cholesterol, triglycerides, fasting plasma glucose, HbA_1c_, prothrombin time, and Mac-2 binding protein glycosylation isomer. All blood samples, including those required for measurement of the serum zinc level, were collected in the early morning after an overnight fast.

### Statistical analysis

Continuous variables were assessed for normal distribution and are expressed as the mean ± standard deviation or median [interquartile range], as appropriate. Patient characteristics were compared within each group using the Wilcoxon signed-rank test. A linear mixed-effects model was used to evaluate longitudinal changes in serum zinc levels, with time as a fixed effect and individual patients included as a random effect to account for within-subject correlations across repeated measurements. Effect sizes (β coefficients), 95% confidence intervals, and corresponding *P-*values were estimated for each time point compared with baseline. Post hoc pairwise comparisons were then performed using marginal estimates derived from the mixed-effects model. All statistical analyses were performed using Stata ver. 15 (StataCorp, College Station, TX). A *P*-value of < 0.05 was considered statistically significant.

## Results

### Patients

Twenty-six patients (17 men, 9 women) of mean age 56.3 ± 12.6 years were enrolled in the study and followed for 36 months. None of the patients took a zinc supplement during the observation period. At baseline, the serum zinc level was < 80 µg/dL in 11 patients and ≥ 80 µg/dL in 15. Twenty-one patients were classified as not having cirrhosis (serum zinc < 80 µg/dL in 9 and ≥ 80 µg/dL in 12) and five as having cirrhosis (serum zinc < 80 µg/dL in 2 and ≥ 80 µg/dL in 3). No patient was found to have a portosystemic shunt on computed tomography or ultrasonography. The baseline characteristics are summarized in Table 1.

**Table 1:** Demographic, clinical, and biochemical data

Variable	Value^†^
	MASH
Number	26
Sex (M/F)	17/9
Age (years)	56 ± 13
Body mass index (kg/m^2^)	29.2 ± 4.2
Aspartate aminotransferase (U/L)	45.9 ± 19.0
Alanine aminotransferase (U/L)	66.7 ± 40.0
Albumin (g/dL)	4.4 ± 0.4
GGT (U/L)	68.4 ± 38.9
Total bilirubin (mg/dL)	0.8 ± 0.4
Platelet count (×10^4^/µL)	23.2 ± 7.4
HDL-C (mg/dL)	46.4 ± 8.5
LDL-C (mg/dL)	131.7 ± 27.6
Triglycerides (mg/dL)	223.0 ± 150.3
Fasting plasma glucose (mg/dL)	139.4 ± 31.0
HbA_1c_ (%)	6.7 ± 1.0
Prothrombin time (% of normal)	126.4 ± 17.3
M2BPGi	0.92 ± 0.41
FIB-4 index	1.73 ± 1.38
Serum Zn level (µg/dL)	82.1 ± 10.3
Patients with Zn < 80 µg/dL/≥80 µg/dL, n	11/15
Average Zn level (< 80 µg/dL/≥80 µg/dL)	72.3/89.3
Without/with cirrhosis, n	21/5

^†^Expressed as the mean ± standard deviation or number of patients as appropriate

*F* Female, *FIB-4* Fibrosis-4, *GGT* Gamma glutamyl transferase, *HbA*_1c_, Glycated hemoglobin, *HDL-C* High-density lipoprotein cholesterol, *LDL-C* Low-density lipoprotein cholesterol, *M* Male, *M2BPGi* Mac-2 binding protein glycosylation isomer, *MASH* Metabolic dysfunction-associated steatotic hepatitis

### Changes in laboratory and clinical parameters

Changes in serum zinc levels following pemafibrate therapy.

#### Overall cohort

**Fig. 1:**
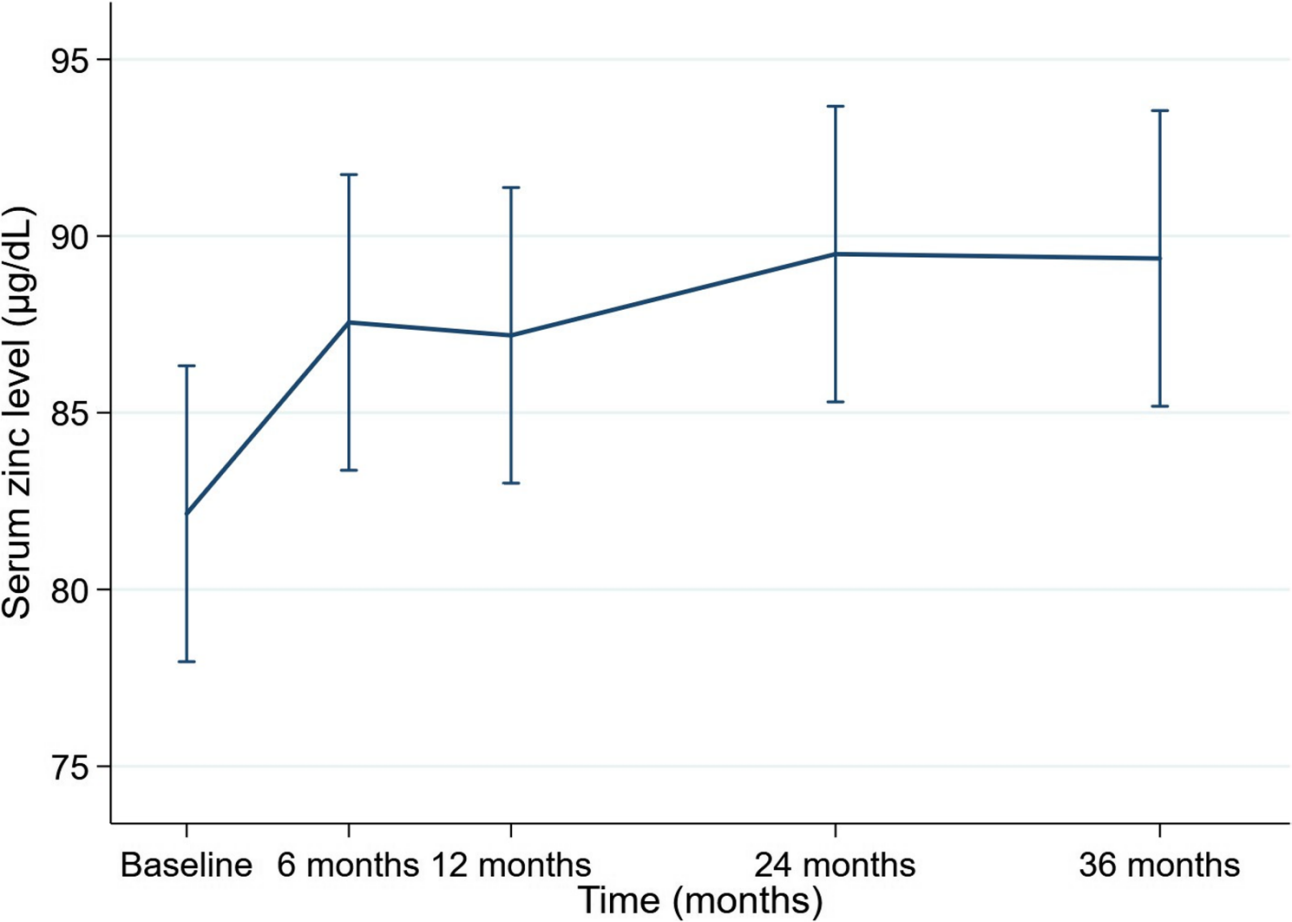
Longitudinal changes in serum zinc levels estimated by a linear mixed-effects model. Estimated marginal means of serum zinc levels increased significantly from 6 months after initiation of pemafibrate therapy and remained elevated throughout the 36-month follow-up period. Values were derived from the linear mixed-effects model with time as a fixed effect and individual patients as a random effect to account for repeated measurements. Error bars represent the 95% confidence intervals

The linear mixed-effects model revealed a significant increase in serum zinc levels over time (*P* < 0.001 for the overall time effect). Compared with baseline, serum zinc levels were significantly higher at 6 months (β = 5.41, 95% confidence interval [CI] 2.05–8.77; *P* = 0.002), 12 months (β = 5.05, 95% CI 1.69–8.40; *P* = 0.003), 24 months (β = 7.35, 95% CI 3.99–10.70; *P* < 0.001), and 36 months (β = 7.22, 95% CI 3.87–10.58; *P* < 0.001) (Fig. 1). These findings are summarized in Table 2.

**Table 2:** Longitudinal changes in serum zinc levels estimated by a linear mixed-effects model

Time point	β coefficient (vs baseline)	95% CI	P-value
6 months	5.41	2.05–8.77	0.002
12 months	5.05	1.69–8.40	0.003
24 months	7.35	3.99–10.70	< 0.001
36 months	7.22	3.87–10.58	< 0.001

Data are presented as β coefficients (effect sizes) with 95% CIs derived from a linear mixed-effects model, with time as a fixed effect and individual patients as a random effect

*CI* Confidence interval

#### Comparison according to cirrhosis status

**Fig. 2:**
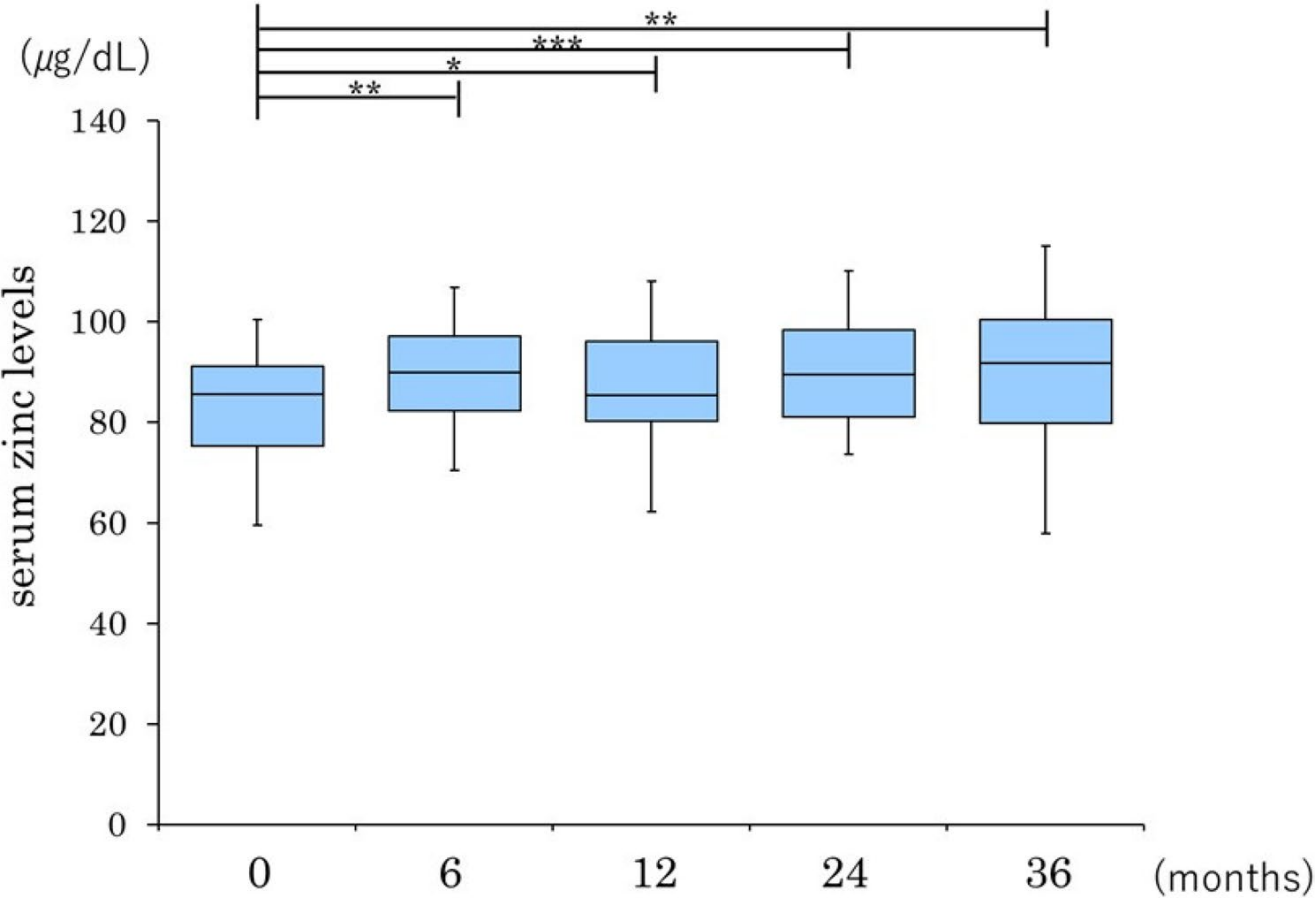
Longitudinal changes in the serum zinc level after pemafibrate therapy in patients with non-cirrhotic MASH. Data are presented as box-and-whisker plots; the box represents the interquartile range, the line inside the box represents the median, and the whiskers indicate the minimum and maximum values. *P < 0.05, **P < 0.01, ***P < 0.001 versus baseline. MASH, metabolic dysfunction-associated steatohepatitis

**Fig. 3:**
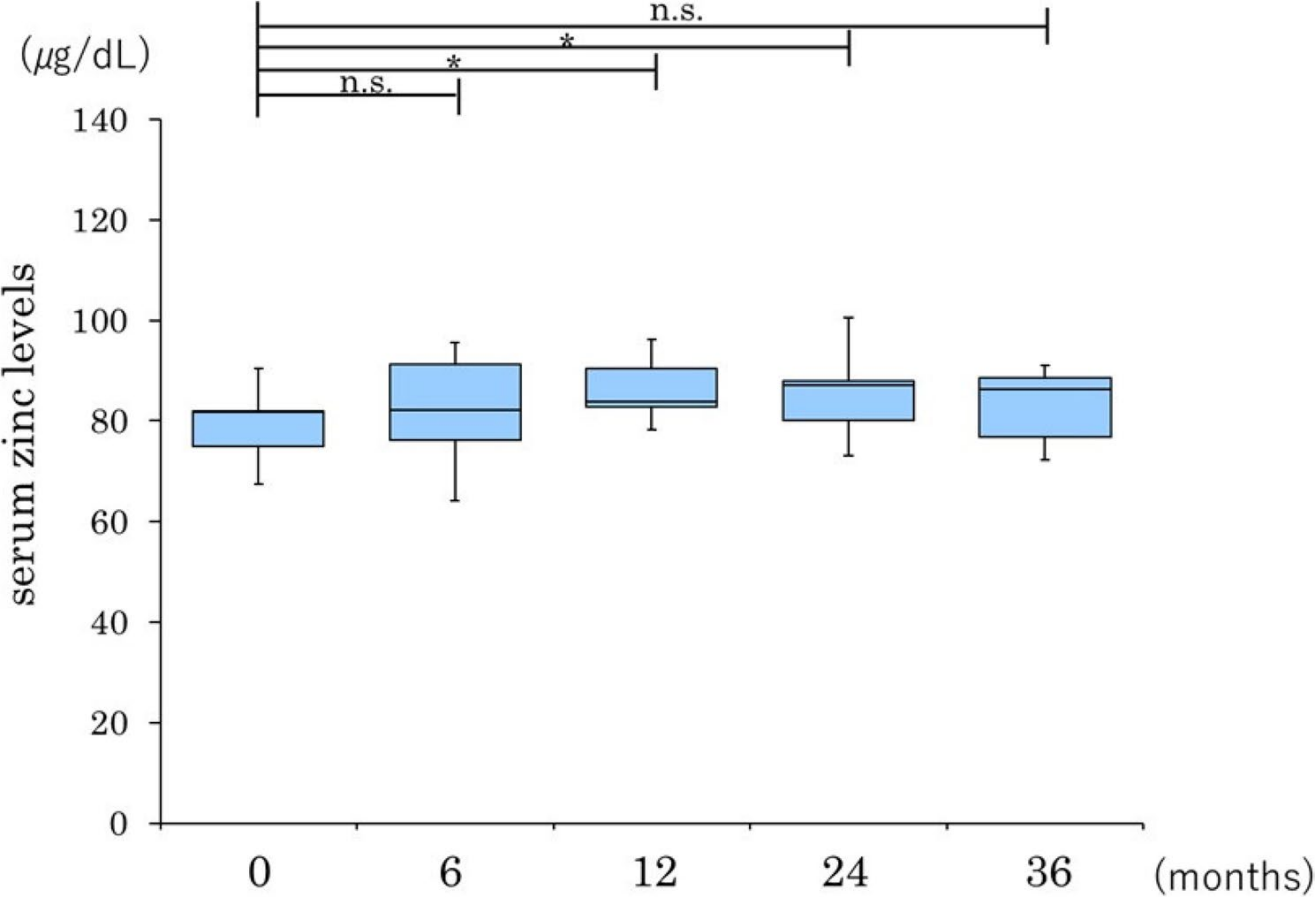
Longitudinal changes in serum zinc levels after pemafibrate therapy in patients with cirrhotic MASH. Data are presented as box-and-whisker plots; the box represents the interquartile range, the line inside the box represents the median, and the whiskers indicate the minimum and maximum values. *P < 0.05 versus baseline, n.s., not statistically significant. MASH, metabolic dysfunction-associated steatohepatitis

In the group without cirrhosis, serum zinc levels showed a significant increase from 6 months after initiation of treatment onwards and remained significantly higher than at baseline throughout the 36-month observation period. In the group with cirrhosis, there was no significant increase at 6 months; however, a significant elevation was noted at 12 months, which persisted until 24 months. By 36 months, serum zinc levels were no longer significantly different from those at baseline (Figs. 2 and 3). However, given the very small number of patients with cirrhosis (*n* = 5), these subgroup findings should be interpreted as exploratory.

#### Comparison according to serum zinc level at baseline

**Fig. 4:**
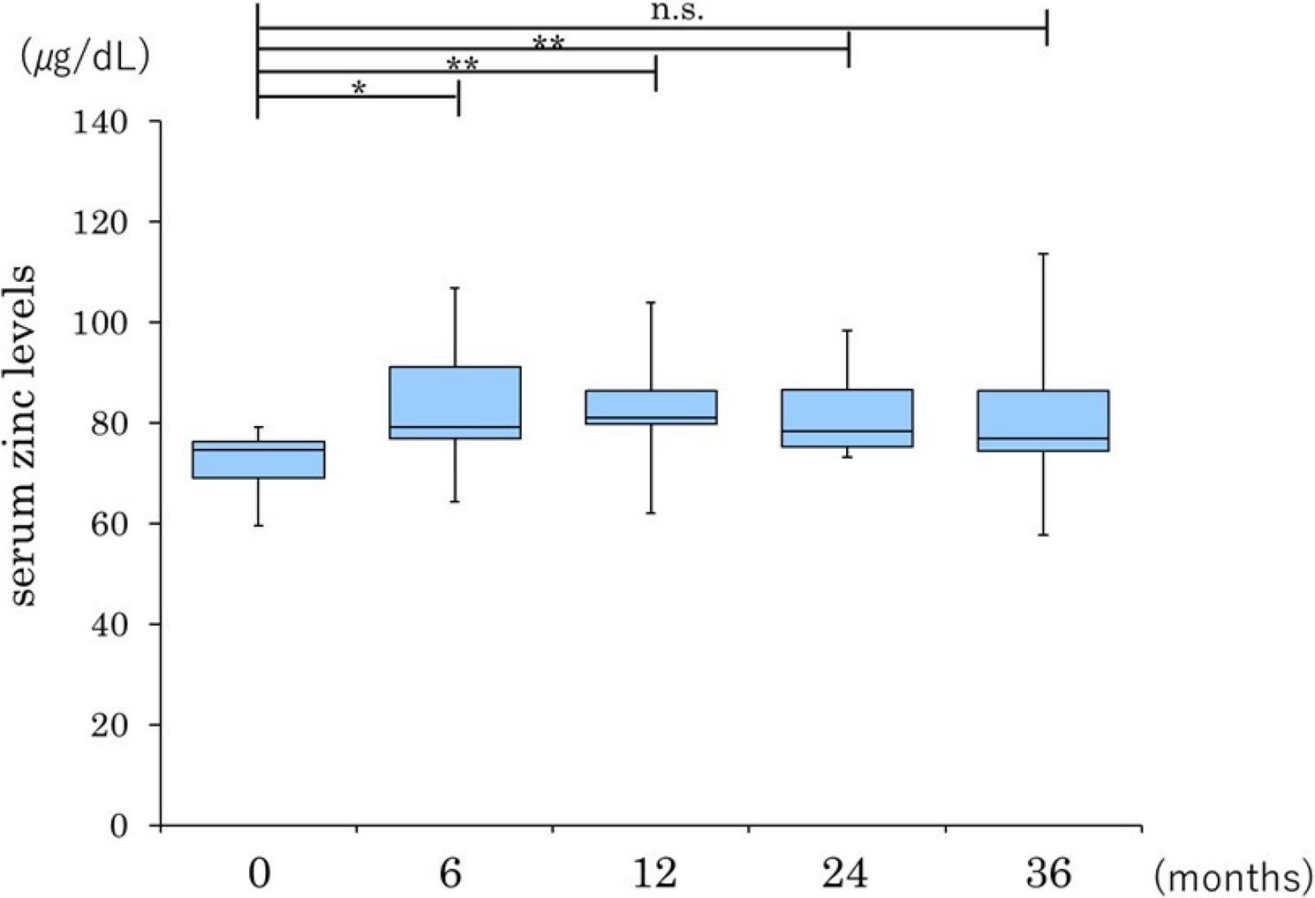
Longitudinal changes in serum zinc levels after pemafibrate in patients with MASH and a baseline zinc level < 80 µg/dL. Data are presented as box-and-whisker plots; the box represents the interquartile range (IQR), the line inside the box represents the median, and the whiskers indicate the minimum and maximum values. *P < 0.05, **P < 0.01 versus baseline, n.s., not statistically significant. MASH, metabolic dysfunction-associated steatohepatitis

**Fig. 5:**
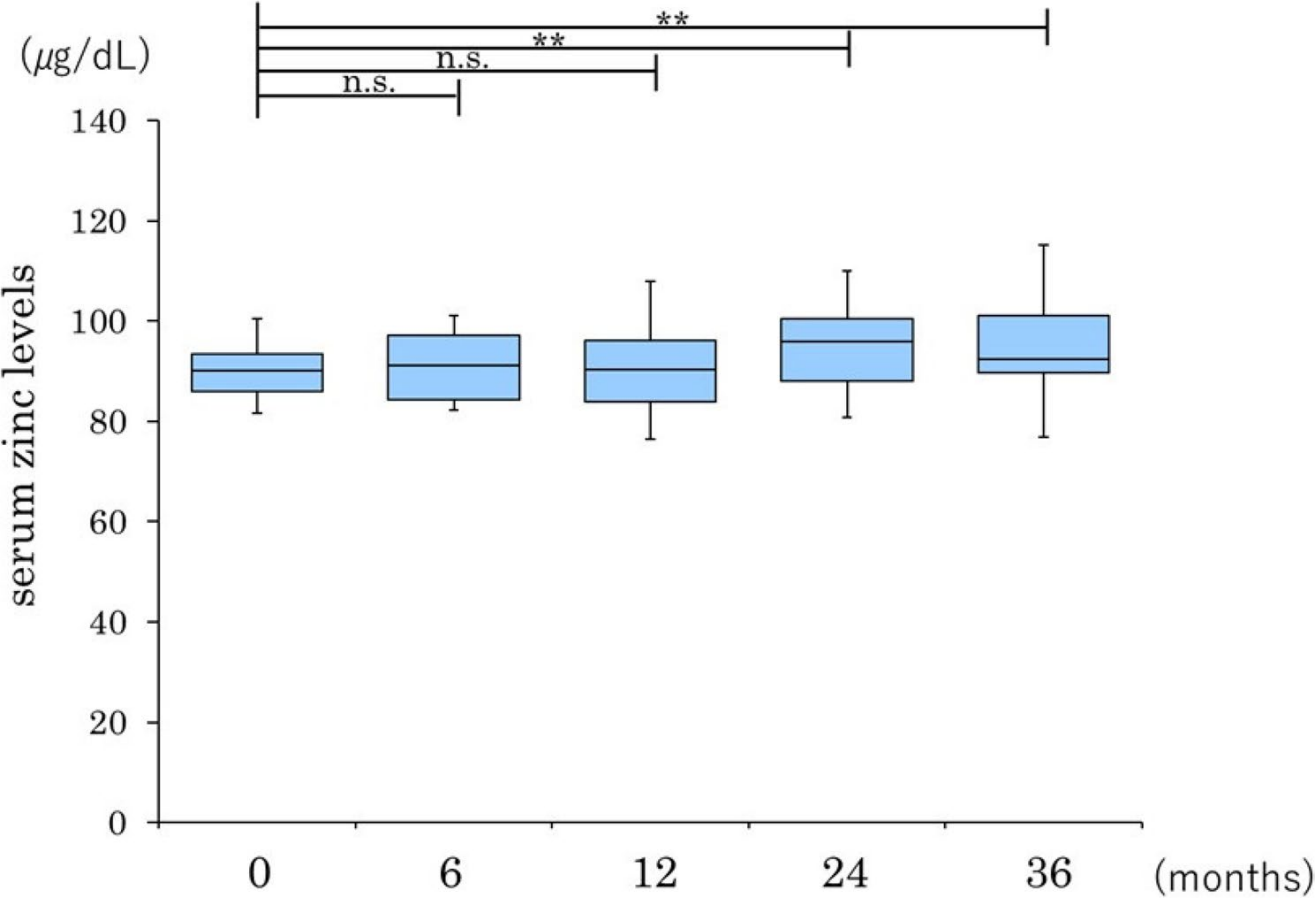
Longitudinal changes in serum zinc levels after pemafibrate in patients with MASH and a baseline zinc level ≥ 80 µg/dL. Data are presented as box-and-whisker plots; the box represents the interquartile range, the line inside the box represents the median, and the whiskers indicate the minimum and maximum values. **P < 0.01 versus baseline, n.s., not statistically significant. MASH, metabolic dysfunction-associated steatohepatitis

There was a significant increase in the serum zinc level at 6 months in patients with a baseline zinc level of < 80 µg/dL, which persisted until 24 months, although the difference was no longer significant at 36 months. Patients with a baseline serum zinc level of ≥ 80 µg/dL showed a significant increase from 24 months onwards, which remained significant at 36 months (Figs. 4 and 5). However, considering the small sample size and limited statistical power after subgroup stratification, these analyses should also be considered exploratory.

### Changes in other laboratory parameters

**Fig. 6:**
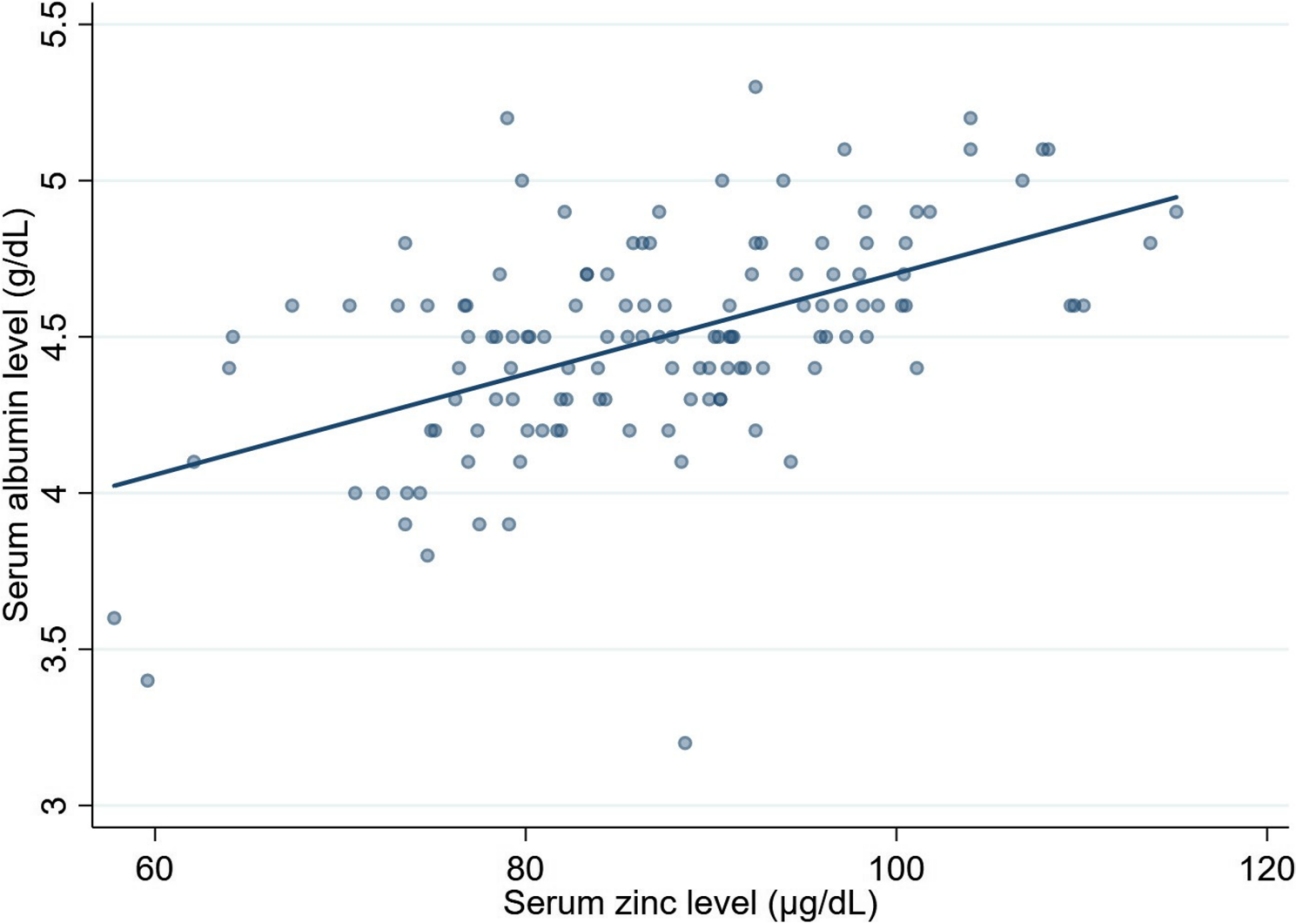
Scatter plot showing the correlation between serum albumin and zinc levels. Each point represents an individual observation. The solid line represents the linear regression fit. A moderate positive correlation was observed (*r* = 0.531, *P* < 0.001)

**Fig. 7:**
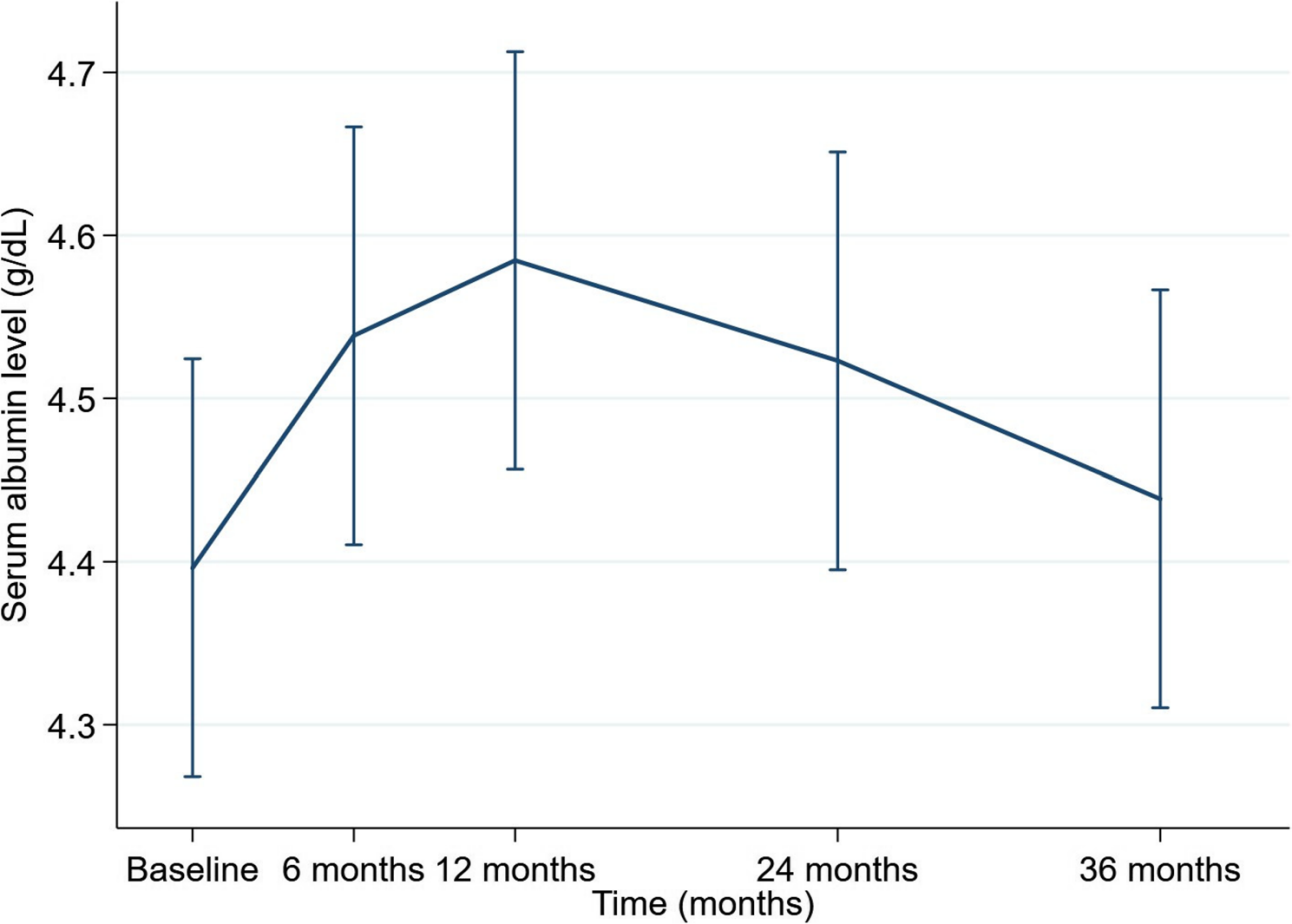
Longitudinal changes in serum albumin levels estimated by a linear mixed-effects model. Serum albumin levels increased significantly during the early phase of treatment, reaching a peak at 12 months, followed by a gradual decline, with no significant difference from baseline at 36 months. Values were estimated using a linear mixed-effects model with time as a fixed effect and individual patients as a random effect. Error bars represent the 95% confidence intervals

When laboratory values at baseline were compared with those at 36 months after initiation of pemafibrate therapy, serum triglyceride levels showed a significant reduction (*P* < 0.01), as did body mass index (*P* < 0.01, aspartate aminotransferase (*P* < 0.01), alanine aminotransferase (*P* < 0.01), gamma-glutamyl transferase (*P* < 0.01), high-density lipoprotein cholesterol (*P* < 0.05), low-density lipoprotein cholesterol (*P* < 0.01), fasting plasma glucose (*P* < 0.05), and Mac-2 binding protein glycosylation isomer (*P* < 0.01). In contrast, although serum albumin did not show a significant difference at 36 months compared with baseline, a linear mixed-effects model analysis demonstrated that serum albumin levels were significantly increased at 6, 12, and 24 months after initiation of pemafibrate therapy (all *P* < 0.05) but not at 36 months (*P* = 0.398), with no significant change in total bilirubin, platelet count, HbA1c, prothrombin time percentage, or the Fibrosis-4 index (Table 3). Considering that circulating zinc is predominantly bound to albumin, we further examined longitudinal changes in the serum albumin level during pemafibrate therapy. A moderate but statistically significant positive correlation was observed between serum albumin and zinc levels (*r* = 0.531, *P* < 0.001) (Fig. 6). These findings indicate that serum albumin levels increased transiently during the early phase of treatment but did not remain significantly elevated at 36 months, in contrast to the sustained increase observed in serum zinc levels (Fig. 7). The findings derived from the linear mixed-effects model are summarized in Table 4.

**Table 3:** Comparison of clinical and biochemical data between baseline and at 36 months

Variable	Value^†^		
	Baseline	36 months	*P*-value
Body mass index (kg/m^2^)	29.2 ± 4.2	28.1 ± 4.0	**
Aspartate aminotransferase (U/L)	45.9 ± 19.0	30.5 ± 11.5	**
Alanine aminotransferase (U/L)	66.7 ± 40.0	25.6 ± 12.0	**
Albumin (g/dL)	4.4 ± 0.4	4.4 ± 0.4	0.3633
GGT (U/L)	68.4 ± 38.9	34.0 ± 17.5	**
Total bilirubin (mg/dL)	0.8 ± 0.4	0.7 ± 0.2	0.5922
Platelet count (×10^4^/µL)	23.2 ± 7.4	23.4 ± 7.1	0.6282
HDL-C (mg/dL)	46.4 ± 8.5	43.8 ± 11.1	*
LDL-C (mg/dL)	131.7 ± 27.6	110.7 ± 29.3	**
Triglycerides (mg/dL)	223.0 ± 150.3	142.7 ± 96.9	**
Fasting plasma glucose (mg/dL)	139.4 ± 31.0	126.7 ± 25.9	*
HbA_1c_ (%)	6.7 ± 1.0	6.8 ± 0.9	0.2813
Prothrombin time (% of normal)	126.4 ± 17.3	120.1 ± 24.2	0.0656
M2BPGi	0.92 ± 0.41	0.73 ± 0.40	**
FIB-4 index	1.73 ± 1.38	1.81 ± 1.28	0.6938

^†^Values are expressed as the mean ± standard deviation or number of patients as appropriate

P-values for continuous parameters were calculated using the Wilcoxon signed-rank test

*FIB-4* Fibrosis-4, *GGT* Gamma glutamyl transferase, *HbA*_1c_ Glycated hemoglobin, *HDL-C* High-density lipoprotein cholesterol, *LDL-C* Low-density lipoprotein cholesterol, *M2BPGi* Mac-2 binding protein glycosylation isomer

*P < 0.05, **P < 0.01

**Table 4:** Longitudinal changes in serum albumin levels estimated by a linear mixed-effects model

Time point	β coefficient (vs baseline)	95% CI	P-value
6 months	0.142	0.044–0.240	0.004
12 months	0.188	0.090–0.286	< 0.001
24 months	0.127	0.029–0.225	0.011
36 months	0.042	−0.056, 0.140	0.398

Values are presented as effect sizes (β coefficients) derived from a linear mixed-effects model with time as a fixed effect and individual patients as a random effect to account for within-subject correlations across repeated measurements

*CI* Confidence interval

## Discussion

A detailed analysis of approximately 2,000 patients with chronic liver disease in Japan found that nearly 90% of patients with cirrhosis had a serum zinc level below 80 µg/dL, while approximately 76% of those without cirrhosis also had a zinc level below this threshold, indicating a high prevalence of hypozincemia in the population with chronic liver disease [24]. Consistent with these results, the present study found that 43% of patients without cirrhosis (9/21) and 40% of those with cirrhosis (2/5) had a serum zinc level below 80 µg/dL before initiation of pemafibrate therapy. Using a linear mixed-effects model to account for repeated measurements, we demonstrated that serum zinc levels increased significantly over time, with sustained elevations observed from 6 months through 36 months after initiation of pemafibrate therapy. These results suggest that pemafibrate therapy is associated with an increase in serum zinc levels even in the absence of zinc supplementation, which is the primary novel finding of this study.

Zinc deficiency in patients with MASH has been reported to induce mitochondrial oxidative stress in hepatocytes, thereby promoting iron overload, exacerbating insulin resistance, and accelerating hepatic fibrosis [11]. Furthermore, zinc has been shown to reduce luminal endotoxin levels and decrease endotoxin-producing bacteria in the intestine, potentially preventing endotoxemia [25]. Collectively, these findings indicate that increasing the serum zinc level may have therapeutic benefits in patients with MASH. In addition, our longitudinal analysis showed a clear increase in serum zinc levels soon after treatment initiation that was maintained over the long term, suggesting a consistent temporal association between pemafibrate therapy and zinc elevation. Furthermore, subgroup analysis revealed that patients without cirrhosis had a sustained increase in serum zinc levels from 6 months after initiation of pemafibrate therapy through to the end of follow-up at 36 months. In contrast, the increase in serum zinc levels was delayed and transient in patients with cirrhosis. However, given the very small sample size in our cirrhosis group (*n* = 5), these findings should be interpreted with caution and regarded as exploratory. When patients were stratified according to their baseline serum zinc level (< 80 µg/dL or ≥ 80 µg/dL), those with a lower baseline zinc concentration showed a significant increase as early as 6 months after initiation of pemafibrate. In contrast, patients with a relatively preserved baseline zinc level (≥ 80 µg/dL) showed a significant elevation starting at 24 months. As with the cirrhosis subgroup analysis, these stratified analyses had limited statistical power and should be interpreted as exploratory rather than as providing definitive evidence. These findings suggest that hepatic functional reserve and baseline zinc status may influence the responsiveness of serum zinc levels to pemafibrate. In addition to the changes in serum zinc and albumin levels, we evaluated longitudinal changes in other clinical and biochemical parameters over the 36-month period. Pemafibrate therapy was associated with significant improvements in serum triglyceride and low-density lipoprotein cholesterol levels. In contrast, high-density lipoprotein cholesterol levels did not improve and instead showed a decrease at 36 months, suggesting that the effect of pemafibrate on high-density lipoprotein cholesterol may be limited in this population. Furthermore, levels of liver enzymes, including aspartate aminotransferase, alanine aminotransferase, and gamma-glutamyl transferase, were significantly reduced, suggesting a potential anti-inflammatory effect of pemafibrate in the liver. Although an improvement in Mac-2 binding protein glycosylation isomer was observed, no significant change was detected in the Fibrosis-4 index. Therefore, the impact of pemafibrate on hepatic fibrosis remains unclear based on the present findings.

Several mechanisms have been proposed to explain zinc deficiency in chronic liver disease, including (a) reduced hepatic synthesis of albumin with disease progression, (b) impaired intestinal absorption of zinc secondary to hypoalbuminemia [26], (c) increased urinary zinc excretion associated with portosystemic shunting in cirrhosis [27], and (d) decreased dietary intake [28].

Several of the above-mentioned mechanisms may have contributed to the observed increase in serum zinc levels in the present study. However, no patients were found to have portosystemic shunting, making a contribution of increased urinary zinc excretion unlikely. Therefore, mechanisms related to the dynamics of albumin, namely, reduced albumin synthesis and impaired intestinal absorption of zinc associated with hypoalbuminemia, are more plausible explanations. Considering that circulating zinc is predominantly bound to serum albumin, the effect of pemafibrate on albumin levels is of particular importance. Moreover, previous studies have reported a strong correlation between serum zinc and albumin levels in patients with chronic liver disease [22, 29, 30]. Therefore, we examined longitudinal changes in serum albumin levels during pemafibrate therapy in greater detail.

Analysis using a linear mixed-effects model revealed that serum albumin levels were significantly increased at 6, 12, and 24 months but not at 36 months. Previous studies have found that pemafibrate therapy leads to a significant increase in serum albumin levels in patients with MASLD as a result of improved hepatic function [31–33]. However, these studies had relatively short treatment durations ranging from 3 to 12 months. In contrast, the present study evaluated patients prospectively over a longer follow-up period of 36 months. We demonstrated that the significant increase in serum albumin levels was maintained for up to 24 months after initiation of pemafibrate, suggesting that elevation of albumin may have contributed to the concomitant increase in serum zinc levels during the early treatment phase. Although a moderate positive correlation between serum albumin and zinc levels was observed, the temporal dissociation between these variables suggests that the increase in serum zinc cannot be fully explained by changes in albumin alone. Importantly, despite the absence of a sustained increase in serum albumin at 36 months, serum zinc levels remained significantly elevated, as demonstrated by the mixed-effects model.

This observation suggests the presence of albumin-independent mechanisms that contribute to zinc elevation in the long term. Potential explanations for the sustained elevation of serum zinc levels at 36 months include the anti-inflammatory and oxidative stress-reducing effects of pemafibrate, which may improve hepatocellular zinc storage capacity, as well as normalization of hepatic zinc transporter expression. However, these mechanistic interpretations remain speculative because inflammatory markers, oxidative stress markers, and zinc transporter expression were not directly evaluated in this study. Therefore, the precise mechanisms underlying these effects remain unclear and warrant further investigation. Furthermore, circulating zinc is not exclusively albumin-bound but is also associated with other proteins such as α2-macroglobulin and transferrin. Moreover, markers of hepatic synthesis and inflammation with a short half-life were not evaluated in this study. Assessment of these biomarkers may help to clarify whether the observed increase in serum zinc reflects changes in hepatic synthesis, inflammation, or redistribution of zinc and should be considered in future studies.

This study has several limitations. First, it was conducted at a single center in a homogeneous population and the sample size was relatively small. Second, this study lacked a comparator group, such as untreated patients or those receiving alternative lipid-lowering therapies. Third, the observation period was limited to 36 months; therefore, longer-term changes in serum zinc levels beyond this period remain unknown. Fourth, although none of the patients initiated zinc supplementation or intentionally consumed zinc-enriched foods during the study period, dietary zinc intake was not quantitatively assessed in detail.　Finally, the generalizability of the present findings to patients without MASH or to other etiologies of chronic liver disease remains uncertain.

## Conclusions

Pemafibrate therapy was associated with a significant increase in serum zinc levels in patients with MASH complicated by hypertriglyceridemia. This longitudinal association was confirmed using a linear mixed-effects model accounting for repeated measurements. However, given the small sample size and absence of a control group, this finding should be interpreted with caution. Further large-scale, controlled studies are required to confirm whether pemafibrate directly modulates zinc metabolism and to clarify the underlying mechanisms.

## Data Availability

The datasets generated and/or analyzed during this study are not publicly available owing to privacy concerns, but are available from the corresponding author upon reasonable request.

## Abbreviations

MASH: Metabolic dysfunction-associated steatohepatitis
MASLD: Metabolic dysfunction-associated steatotic liver disease
NAFLD: Nonalcoholic fatty liver disease
NASH: Nonalcoholic steatohepatitis
